# Understanding Educational and Psychosocial Factors Associated with Alcohol Use among Adolescents in Denmark; Implications for Health Literacy Interventions

**DOI:** 10.3390/ijerph15081671

**Published:** 2018-08-06

**Authors:** Claudia König, Mette V. Skriver, Kim M. Iburg, Gillian Rowlands

**Affiliations:** 1 Department of Public Health, Aarhus University, Bartholins Allé 2, Bygning 1260, 8000 Aarhus C, Denmark; claudiakoe7@gmail.com (C.K.); mette.skriver@ph.au.dk (M.V.S.); kmi@ph.au.dk (K.M.I.); 2 Institute of Health and Society, Newcastle University, NE1 7RU Newcastle upon Tyne, UK

**Keywords:** health literacy, alcohol, adolescent, local context

## Abstract

*Background.* Alcohol misuse is a global public health priority, with a variation in prevalence and impact between countries. Alcohol misuse in adolescence is associated with adverse psychological, social and physical health. Adolescents in Denmark have higher alcohol consumption and problematic alcohol use than adolescents in other European countries. Associations between social determinants of health (SDH), psycho-social factors and alcohol consumption are complex and influenced by national context and cultures. This study explored these associations in Danish adolescents. *Method*. The European School Survey Project on Alcohol and Other Drugs (ESPAD) survey collects data on alcohol and substance use among 15–16-year-old European students. Data contributed by Danish students to the 2011 survey were analyzed. The outcomes of interest were alcohol consumption (any, intoxication and problematic). Health literacy was not directly measured, so self-described educational performance and knowledge about alcohol were used as proxies for health literacy. Exploratory factors thus included socio-demographic, health literacy-related (knowledge about alcohol, educational performance) and psycho-social factors, as well as expectancies of the effect of alcohol (both positive and negative) and self-reported health. Univariate and multivariate logistic regression analyses were undertaken. *Results*. Of the 2768 adolescents who participated in the survey, 2026 (80%) consumed alcohol during the last 30 days, 978 (38%) were intoxicated at least once during the last 30 days, and 1050 (41%) experienced at least one problem because of alcohol use during the last 12 months. Multivariable analysis showed that the factors associated with higher alcohol intake were gender, poor relationships with parents, expectancies of the impact of alcohol (both positive and negative), and the influence of peers and their alcohol use. Higher school performance was related to lower alcohol consumption. Low socio-demographic status was not associated with higher alcohol consumption. *Conclusions*. This study confirmed the high levels of alcohol intake, intoxication, and problem drinking amongst the Danish students in the survey and the complexity of the socio-demographic, psychosocial, health literacy-related, and environmental factors associated with alcohol behaviours. Approaches to addressing the issue of alcohol use in Danish adolescents will need to be multi-factorial, including supporting students to develop alcohol-related health literacy skills to enable them to make informed choices.

## 1. Introduction

Alcohol is one of the world’s top three priority areas in public health and is the third largest risk factor for disease and early death in Europe [1,2,3]. In childhood and adolescence, alcohol use is associated with adverse psychological, social and physical health consequences, including violence, accidents, injury, risky sexual behaviour, academic failure and increased risk of the use of other substances [4,5]. Alcohol may interfere with adolescent brain development and functioning [6]. Those who are early initiators, excessive drinkers and who have multiple risk behaviours are especially likely to experience adverse health outcomes [7]. Alcohol use in adolescence has been associated with alcohol consumption and health issues in later life [4,8,9].

Alcohol use in adolescence is common in many European countries [8,10,11,12], particularly so in Denmark with high levels of lifetime alcohol consumption (92%), alcohol consumption during the last 30 days (73%), ‘heavy episodic drinking’ (drinking more than five drinks on the same occasion) during the last 30 days (56%), and ‘intoxication’ during the last 30 days (32%) [12]. The minimum legal age of alcohol consumption in Denmark is 16 years.

The HBSC study on adolescents’ health showed social inequalities for a number of health outcomes, with higher socioeconomic resources in general being associated with better health outcomes and positive social contexts with respect to family, peers and school [8,10]. However, associations between social determinants of health (SDH: the conditions in which people are born, grow, live, work and age [13]) and risk behaviors such as alcohol consumption are often more complex, and show contradictory results [8,10,14,15,16,17,18,19,20,21]. Additional factors increasing the complexity of understanding the reasons underlying alcohol consumption in adolescence are the associations with psycho-social factors such as social relationships to parents and friends [14,17,22,23]. The variation in alcohol consumption in adolescents between countries [12] is likely to reflect differences in environmental (i.e., national, cultural and legal) contexts. 

Health literacy, ‘the motivation, knowledge and competencies to access, understand, appraise and apply health information in order to make judgments and take decisions in everyday life concerning healthcare, disease prevention and health promotion to maintain or improve quality of life throughout the course of life’ [24], may be another factor of importance in enabling informed alcohol consumption. Health literacy is gaining increasing focus internationally, as it empowers individuals and communities through enhancing capacities for promoting health and preventing and managing disease. It is seen as key to the delivery of health promotion strategies [25]. Health literacy can be viewed at several levels of cognitive capacities. Functional skills are the reading and writing skills needed to be able to function effectively in everyday situations; communicative/interactive skills are more advanced cognitive and literacy skills which, together with social skills, can be used to actively participate in everyday activities, to extract information and derive meaning from different forms of communication, and to apply new information to changing circumstances; and critical skills are more advanced cognitive skills which, together with social skills, can be applied to critically analyze information and to use this information to exert greater control over life events and situations [26]. It is being increasingly recognized that health literacy may be important in understanding—and developing interventions to address—risky alcohol consumption. ‘Alcohol health literacy’ has been defined as is ‘the degree to which individuals have the capacity to obtain, process and understand knowledge about alcohol content, units, strengths and harms’ [27]. This definition thus fits within ‘functional health literacy’ as described by Nutbeam [26]. At present, no wider definitions of alcohol health literacy (i.e., incorporation of the concept of interactive and critical alcohol health literacy) exist.

In summary, alcohol behavior in adolescents is an important cause of health, educational, legal and social problems. The reasons underlying alcohol consumption are complex and in addition are likely to be influenced by national and local alcohol-related contexts and cultures and health literacy. This study was an in-depth exploration of these associations in Denmark, a country with high levels of alcohol use in adolescents.

## 2. Materials and Methods

### 2.1. Design, Data Collection and Participants

Data were collected in 2011 by the European School Survey Project on Alcohol and Other Drugs (ESPAD), a project aiming to collect comparable data on substance use among 15–16-year-old European students [11]. Analyses were performed on Danish data. The study population were students in 9th grade public and private schools in Denmark, primarily aged 15–16 years, who participated in the 2011 ESPAD survey. Ninety-seven schools with a total of 2768 Danish students participated. The ESPAD report provides details on survey methodology and data quality [11].

### 2.2. Aim

The study aimed to explore associations between social determinants of health including health literacy as indicated by educational performance and knowledge about the risks of high levels of alcohol intake, psycho-social factors and alcohol consumption in Danish adolescents, in order to give recommendations for action to support safer alcohol consumption in this age-group.

### 2.3. Measures

For the analyses, the research team selected variables likely to be relevant, given published research in the area. Measures used in the analyses are described below. The test-retest reliability of the measurements used in ESPAD have been described [28]. Reliability has been described both within and between countries. Validity (in this case, the degree to which the survey measures what it is intended to measure, namely substance use in students) is high; whilst direct measures of alcohol and drug consumption (e.g., through saliva or urine sampling) are not taken, in post-survey interviewing and questionnaires, students have consistently reported truthful responses in earlier survey completions.

Health literacy was not directly measured in the survey. We thus identified those variables that mapped most closely onto the definition of health literacy; i.e., cognitive skills and alcohol knowledge [26,27]. Cognitive skills were captured through student educational performance, whilst alcohol knowledge was evaluated through student knowledge that consuming 4–5 alcoholic drinks a day is risky behavior. Students’ educational performance was measured through self-report using the Danish education system grading system, categorized into ‘below average’ (grade less than 6), ‘average’ (grade 6–8,), and ‘above average’ (grade 9 or higher).

### 2.4. Socio-Demographic Factors

These included gender, age, educational attainment of the parent(s), and socio-economic status (SES). Parental educational attainment was grouped into low (lower secondary education), medium (upper secondary education), and high (medium long or higher education). SES was assessed by the perceived financial situation of the adolescents’ family compared to other families in Denmark and grouped into three categories: ‘better off’, ‘about the same’, and ‘less well off’.

### 2.5. Psycho-Social Factors

Respondents were asked how satisfied they usually were with their relationship with their mother, father and friends. Response categories ranged from ‘very satisfied’ to ‘not at all satisfied’ and answers were grouped into two categories: ‘satisfied’ and ‘neither nor/not satisfied’. Respondents also reported how often during the last 12 months they had experienced serious problems with their parents and friends, categorized into never (0) and ‘1 or more occasions’.

Parental monitoring was measured by students’ assessment of parents’ knowledge of their whereabouts at weekends, grouped into two categories: ‘know always/know quite often’ and ‘know sometimes/usually don’t know’.

Peer alcohol consumption was measured through variables including both the alcohol consumption of friends and siblings. Respondents estimated how many of their friends drink alcoholic beverages or get drunk. The answers were categorized into ‘none/a few/some’ and ‘most/all’). In addition, the students reported whether any of their older siblings drink alcoholic beverages or get drunk (‘yes’ or ‘no’).

### 2.6. Other Factors

Students’ well-being and their knowledge related to alcohol consumption were included. Respondents rated their satisfaction with their own health and separately with themselves and their lives. Answers were categorized into ‘satisfied’ and ‘neither nor/not satisfied’. Respondents also appraised the extent to which people risk harming themselves (physically or in other ways) if they have four or five drinks nearly every day (categorized: ‘don’t know/no risk’ and ‘risk’). Alcohol expectancies were measured by asking the students how likely it was that each of the following things would happen to them personally if they drank alcohol. Their answers were grouped into positive and negative alcohol expectancies as follows and categorized into ‘likely’ and ‘unsure/unlikely’. Positive alcohol expectancies included feeling relaxed, feeling happy, feeling more friendly and out-going, having a lot of fun, and forgetting their problems. Negative alcohol expectancies included getting into trouble with police, harming their health, not being able to stop drinking, getting a hangover, doing something they would regret, feeling sick.

### 2.7. Alcohol Consumption

Outcome variables were chosen to reflect a gradient in severity of alcohol consumption i.e., (1) alcohol consumption during the last 30 days; (2) intoxication during the last 30 days; and (3) problems because of own alcohol use during the last 12 months. Alcohol consumption was measured through the number of occasions (if any) in which respondents had any alcoholic beverage to drink during the last 30 days; intoxication was measured through the number of occasions (if any) respondents had been intoxicated during the last 30 days; and ‘problems from drinking’ was measured through the number of occasions respondents had experienced one the following during the last 12 months because of their alcohol use: physical fights, accident or injury, serious problems with parents, serious problems with friends, performing poorly at school or work, being victimized by robbery or theft, trouble with police, being hospitalized or admitted to an emergency room, engaging in sexual intercourse without a condom, or engaging in sexual intercourse that was regretted the next day. The answers of all three outcome variables were categorized into never (0) and ‘1 or more occasions’.

### 2.8. Statistical Analysis

Initially, descriptive analyses of relevant variables were conducted. Following this, the relations between alcohol consumption and SDH, HL and psycho-social factors were studied using logistic regression analyses, both univariable and multivariable.

Univariable analyses explored the associations of explanatory variables (SDH, HL, psycho-social factors) and (1) alcohol consumption; (2) intoxication; and (3) problem drinking.

Multivariable analyses were then undertaken including all explanatory variables. To simplify the analysis, only one variable representing the students’ school performance, i.e., average grade, was included.

Three sets of analyses were carried out, i.e., associations between SDH, HL, psycho-social factors and (1) alcohol consumption; (2) intoxication; and (3) problem drinking.

As a Supplementary Materials analysis, the dataset was divided by gender and all above described analyses were conducted to determine if results differed by gender.

All statistical analyses were performed using STATA (14.1). The level of significance was set at *p* < 0.05.

## 3. Results

### 3.1. General Characteristics

The sample contained responses from 2768 students, with approximately equal numbers of male and female students. Data on key variables are shown in Table 1. It should be noted that not all students answered every question.

Of note is the high levels of alcohol intake in the last 30 days (*n* = 2026, 79.64%), intoxication (*n* = 978, 38.38%), and experience of problems as a result of drinking alcohol (*n* = 1050, 41.18%), and high levels of alcohol exposure from peers (*n* = 2410, 87.8%) and siblings (*n* = 1632, 87.41%). A very high proportion of the students expected positive outcomes to arise from drinking alcohol (*n* = 2341, 92.79%), with 686 of students (27.32%) reporting that they expected not to experience negative consequences from drinking alcohol. When socio-economic factors are considered, whilst nearly one third (*n* = 606, 31.88%) came from families with low paternal education, only 283 (10.49%) felt that their family was ‘less well off than average’. Many students reported negative family and peer relationships, including serious problems with either parent (*n* = 752, 27.41%) and/or friends (*n* = 896, 32.68%).

Only a very small number (115 students, 4.2%) did not recognize the fact that drinking 4–5 alcoholic drinks a day brought risks.

### 3.2. Univariable Analysis

Associations between explanatory variables (SDH, HL, psycho-social factors) and all three outcome variables (alcohol consumption, intoxication, problem drinking) from univariable logistic regression analyses can be accessed in Table S1 in the Supplementary Materials.

### 3.3. Multivariate Analyses

Table 2 provides an overview of the results of the multivariable analyses.

The variables indicating an increased risk of any alcohol consumption and more risky alcohol consumption (recent intoxication or having ever experienced problems due to drinking) were male gender, consumption of alcohol by friends and experiencing problems with friends. Those whose friends drink any alcohol were more likely to drink alcohol themselves, but not to engage in more risky drinking, whilst those whose friends become intoxicated were more likely to engage in risky drinking themselves. In addition, those students who reported having experienced problems with peers were more likely to report any alcohol consumption and more risky drinking. Students who reported not being satisfied with the relationship with their mother and having experienced serious problems with parents were more likely to report risky drinking.

Students with poor school performance had higher levels of alcohol consumption and risky drinking, but this did not reach statistical significance. Students who reported their school performance as above average were less likely to have consumed alcohol in the last 30 days and to report problems arising from alcohol consumption. The same result was seen concerning intoxication, although the finding was not statistically significant; overall, a non-significant trend was seen whereby those with above-average self-reported school performance had a lower likelihood of drinking, intoxication and problems arising from alcohol use.

As might be expected, students who expected positive outcomes from alcohol drinking were more likely to drink at all and to exhibit risky drinking. In contrast, understanding the risk of high alcohol intake and having expectancies of negative outcomes of drinking alcohol showed the opposite effects to those expected. Almost all the students (95.8%) knew that consuming 4–5 drinks a day has health risks; knowledge of these risks was associated with an increased likelihood of having drunk any alcohol in the last 30 days, although not with reporting more risky drinking. Students with expectations of negative outcomes were more likely to have undertaken more risky drinking, but not more likely to have drunk at all in the last 30 days.

Parents’ socioeconomic status (education and wealth) was not associated with either any alcohol consumption in the last 30 days or more risky drinking behavior.

### 3.4. Gender-Separated Analysis

Supplementary Materials analyses by gender were conducted. Results were similar and are therefore not described in detail here. Results are accessible in Table S2 in the Supplementary Materials.

## 4. Discussion

### 4.1. Key Results

Alcohol drinking in 15–16-year-olds in Denmark is very high, with nearly 80% of students having drunk alcohol in the last 30 days, and approximately 40% describing risky drinking i.e., intoxication in the last 30 days or ever having experienced problems due to drinking. Danish adolescents are living in a high-alcohol environment, with over 80% of students reporting that their peers drink and, for those reporting drinking in siblings, over 80% reporting that their siblings drink.

Drinking alcohol is more likely in those students who are male, who are in a peer group that drinks alcohol, and for those experiencing problems with friends and at home. Expecting positive outcomes from drinking alcohol is associated with more drinking, but, surprisingly, expecting negative outcomes from alcohol and being aware that high alcohol consumption is risky were also associated with more drinking. Knowledge of the risks of high alcohol consumption, which was almost universal, was associated with higher likelihood of drinking any alcohol but not of undertaking riskier drinking.

Whilst poor school performance was not statistically significantly associated with alcohol consumption and risky drinking, students reporting above average school performance were less likely to drink at all and to experience problems as a result of drinking.

Family socio-economic status was not associated with alcohol intake.

### 4.2. Strength and Limitations

The data for the statistical quantitative analysis were gathered in 2011 by the European study ‘European School Survey Project on Alcohol and Other Drugs’ (ESPAD), a project that collected extensive data on substance use among adolescents aged 15–16 years. This comprehensive dataset, from a large, well established study with reliable, well validated questions and high-quality data, provides an excellent opportunity to study hazardous alcohol consumption in the Danish setting.

However, a number of limitations need to be recognized when interpreting our results.

Firstly, when examining the associations between social determinants of health, variables used as proxy measures for health literacy, psychosocial factors and alcohol consumption, we considered only variables that were available from the ESPAD survey. Although we included a variety of socioeconomic and psychosocial variables, several other factors may affect alcohol use in adolescents and the influence of the social background may be wider. There may be other important factors that were not included in the study. Secondly, data was entirely self-reported by adolescents and may be subject to various types of biases. Thirdly, health literacy was not directly measured. The variables we used as proxies for health literacy, whilst closely associated with the current definition of alcohol health literacy (knowledge about the risks of alcohol) and interactive and critical skills (educational competence), are not direct measures and thus should be interpreted with caution. Finally, the cross-sectional nature of the data prevents any conclusions of causal relationships between social determinants of health, psychosocial factors and alcohol consumption.

### 4.3. How this Research Links to Current Knowledge

This study supported many of the findings in previous research, in particular the influence of peers on alcohol consumption [14,23], the protective effect of parental emotional support/parenting factors [14,17,22,29], the associations between positive alcohol expectancies and alcohol intake [30], and the weak association between alcohol consumption and socio-economic factors [14,23].

There were some important differences, however, between the findings in this study and previous research. Specifically, previous research from the U.S. has shown that the gender differences in alcohol consumption seen in adults (i.e., men consuming more alcohol and experiencing more alcohol-related problems than females [31]) are not seen in U.S. adolescents [32]. The study described here shows that, in Denmark, the gender pattern of alcohol drinking in adolescents mirrors that seen in adults, with male students drinking more than female students. 

Another difference between the results of this Danish study and previous research is in the associations between negative alcohol expectancies and alcohol drinking. Whereas previous studies have shown that negative alcohol expectancies tend to have the expected associations with alcohol drinking (i.e., more negative alcohol expectancies are associated with lower drinking) [30], the findings from the present study showed the opposite association; expecting negative effects of drinking were associated with more alcohol drinking.

### 4.4. Interpretation

Our study shows that, in Denmark, an environment with high levels of alcohol availability and alcohol consumption, students aged 15 to 16 years who are at risk of high alcohol consumption and risky drinking are more likely to be in a peer group that drinks alcohol and to be experiencing problems with friends and at home. They are not likely to be performing poorly at school, or to come from socio-economically deprived backgrounds.

The results indicate the likely importance of legal and cultural contexts; whilst alcohol consumption at the age of 15 years is not legal in Denmark, it is extremely common, and students have very high exposure to alcohol. This may explain the contrast to, for example, findings for U.S. adolescents, where the legal age of drinking alcohol is much higher (21 years in many U.S. states compared to 16 years in Denmark), and students may have a lower exposure to alcohol from family and friends.

The results relating to health literacy may also reflect the ‘alcohol environment’ in which the students are living. Almost all (95.8%) of Danish students knew that taking 4–5 alcohol drinks a day is a risk to health; given the ubiquity of alcohol consumption amongst students, it is perhaps not surprising that this knowledge was not associated with less alcohol consumption in general, and it did not appear have any protective effect against riskier drinking. Educational competency, however, which may correspond to Nutbeam’s ‘interactive’ and ‘critical’ health literacy, did appear to be associated with less overall drinking, and also with less risky drinking.

Our findings indicate that interventions to support 15 to 16-year-olds in Denmark to develop skills to make informed choices about alcohol consumption will need to be multifaceted. Any interventions need to consist of more than simply giving information about alcohol and its effects and developing skills to find and understand information about alcohol (functional health literacy). The finding that expecting negative outcomes from drinking and being aware of risks from high alcohol intake are both associated with a higher risk of drinking indicate that more complex issues are at play. The finding that students in alcohol-drinking peer groups are more likely to drink alcohol, as are students experiencing problems with friends and at home, together with the apparent protective effects of high education competence, indicate that approaches that develop wider cognitive competencies together with self-confidence in resisting peer pressure and ways to manage stressful situations within and outside the home, (i.e., critical alcohol health literacy—skills to critically analyze information and to use this information to exert greater control over alcohol drinking) should be the focus of interventions in this age group. Before such interventions can be developed, qualitative studies need to be undertaken with students themselves to better understand students’ experiences, and to gather their insights on what approaches might help.

Finally, it is likely that the availability of alcohol, cultural issues, and legal frameworks in relation to alcohol will also play a part. It may be that the risk factors and socio-demographic associations will differ in different national settings and amongst different cultural groups.

## 5. Conclusions

In Danish society, adolescents have a high exposure to and cultural acceptance of alcohol drinking. In 15 to 16-year-olds, alcohol consumption, including riskier drinking patterns, is associated with peer-group exposure to alcohol and experiencing problems with friends and at home, and not with alcohol knowledge and socio-economic deprivation. Higher school performance is associated with lower alcohol drinking, including risky alcohol behaviors. In Danish settings, interventions to give adolescents the capacities to make and enact informed decisions about alcohol should focus on building critical alcohol health literacy—skills to critically analyze information about alcohol drinking and its risks and to use this information to exert greater control over alcohol consumption to reduce the risks of current and future harm—rather than simply focusing on increasing knowledge. In other countries, with different levels of alcohol exposure and availability and different alcohol cultures, the socio-demographic and psycho-social factors, and the impact of knowledge about and expectations of alcohol drinking may be different; analysis of local associations is required to develop interventions that are locally specific and culturally relevant.

## Figures and Tables

**Table 1:** Description of sample and variables of interest.

Total	2768
Male Gender	1319 of 2750 (48.0%)
*Alcohol intake*	
Alcohol taken on ≥ 1 occasion in last 30 days	2026 of 2544 (79.64%)
Alcohol intoxication on ≥ 1 occasion in last 30 days	978 of 2548 (38.38%)
Have experienced problems from alcohol use during the last 12 months	1050 of 2550 (41.18%)
*Alcohol environment*	
Most or all friends drink alcohol	2410 of 2745 (87.80%)
Most or all friends get intoxicated	1927 of 2746 (70.17%)
Older siblings drink alcohol	1632 of 1867 (87.41%)
Older siblings get intoxicated	1436 of 1808 (79.42%)
*Alcohol expectancies and beliefs*	
Reporting that they are likely to experience positive outcomes from alcohol	2341 of 2523 (92.79%)
Reporting that they are unlikely to experience negative outcomes from alcohol	686 of 2511 (27.32%)
*Socio-economic status*	
Highest level of schooling father: Lower secondary education or less	606 of 1901 (31.88%)
Highest level of schooling mother: Lower secondary education or less	360 of 2082 (17.29%)
Families’ wealth: Less well off than average	283 of 2697 (10.49%)
*Health*	
’Not satisfied’ or ‘neither satisfied nor dissatisfied’ with own health	392 of 2739 (14.31%)
’Not satisfied’ or ‘neither satisfied nor dissatisfied’ with themselves	654 of 2735 (23.91%)
*Family and peer relationships*	
’Not satisfied’ or ‘neither satisfied nor dissatisfied’ with maternal relationship	303 of 2712 (11.17%)
’Not satisfied’ or ‘neither satisfied nor dissatisfied’ with paternal relationship	450 of 2635 (17.08%)
Parents know sometimes or usually do not know where respondent is on Saturday nights	148 of 2746 (5.39%)
Have experienced ‘serious problems’ with parents on ≥ 1 occasion	752 of 2744 (27.41%)
’Not satisfied’ or ‘neither satisfied nor dissatisfied’ with relationships with friends	205 of 2740 (7.48%)
Have experienced ‘serious problems’ with friends on ≥ 1 occasion	896 of 2742 (32.68%)
*Health literacy proxies*	
School performance† below average	436 of 2664 (16.37%)
Knowledge that 4–5 drinks a day brings health risks: answered ‘no’ or ’don’t know’	115 of 2740 (4.20%)

**Table 2:** Multivariable analyses.

Explanatory Variable	Drinking any Alcohol during the Last 30 Days	Been Intoxicated during the Last 30 Days	Problems Because of Own Alcohol Use during the Last 12 Months
	OR (95% CI)	OR (95% CI)	OR (95% CI)
Gender			
Male	Reference	Reference	Reference
Female	0.56 (0.37 to 0.84) *	0.79 (0.58 to 1.08)	0.64 (0.46 to 0.88) **
School performance †			
6–8.9 (average)	Reference	Reference	Reference
>9 (above average)	0.60 (0.39 to 0.93) *	0.88 (0.60 to 1.29)	0.58 (0.39 to 0.86) **
<6 (below average)	1.34 (0.67 to 2.69)	1.42 (0.89 to 2.26)	1.22 (0.75 to 1.98)
Father’s education			
Medium long or higher education	Reference	Reference	Reference
Upper secondary education	0.66 (0.40 to 1.09)	1.10 (0.74 to 1.64)	1.19 (0.79 to 1.78)
Lower secondary education or less	0.84 (0.49 to 1.42)	0.96 (0.63 to 1.45)	1.40 (0.92 to 2.13)
Mother’s education			
Medium long or higher education	Reference	Reference	Reference
Upper secondary education	1.39 (.88 to 2.21)	0.90 (0.63 to 1.29)	1.27 (0.88 to 1.84)
Lower secondary education or less	0.97 (0.53 to 1.79)	0.76 (0.48 to 1.22)	1.35 (0.83 to 2.18)
Wealth			
About the same	Reference	Reference	Reference
Better off	1.15 (0.76 to 1.74)	1.33 (0.97 to 1.83)	1.22 (0.88 to 1.70)
Less well off	0.74 (0.35 to 1.58)	0.96 (0.51 to 1.81)	1.10 (0.58 to 2.07)
Pos. alcohol expectancies (things happen to participants personally)			
Likely	Reference	Reference	Reference
Unsure/Unlikely	0.28 (0.14 to 0.56) **	0.25 (0.07 to 0.87) *	0.17 (0.05 to 0.60) **
Neg. alcohol expectancies (things happen to participants personally)			
Unsure/Unlikely	Reference	Reference	Reference
Likely	1.18 (0.77 to 1.81)	2.20 (1.50 to 3.21) **	2.80 (1.90 to 4.12) **
Belief risk			
Risk	Reference	Reference	Reference
No risk or do not know	0.22 (0.08 to 0.55) **	0.57 (0.22 to 1.52)	0.43 (0.16 to 1.18)
Satisfied health			
Satisfied	Reference	Reference	Reference
Not satisfied or neither nor	0.70 (0.40 to 1.23)	0.88 (0.56 to 1.37)	0.94 (0.59 to 1.51)
Satisfied themselves			
Satisfied	Reference	Reference	Reference
Not satisfied or neither nor	1.20 (0.73 to 1.97)	1.36 (0.93 to 1.99)	1.05 (0.71 to 1.57)
Parents know where their children are on Saturday			
Know always/quiet often	Reference	Reference	Reference
Know sometimes/Usually do not know	0.86 (0.27 to 2.74)	0.84 (0.39 to 1.81)	1.03 (0.41 to 2.56)
Satisfied relationship with mother			
Satisfied	Reference	Reference	Reference
Not satisfied or Neither nor	1.38 (0.61 to 3.11)	1.18 (0.69 to 2.04)	1.96 (1.08 to 3.56) *
Satisfied relationship with father			
Satisfied	Reference	Reference	Reference
Not satisfied or Neither nor	0.80 (0.44 to 1.45)	1.17 (0.74 to 1.86)	1.01 (0.62 to 1.63)
Satisfied relationship with friends			
Satisfied	Reference	Reference	Reference
Not satisfied or Neither nor	0.33 (0.17 to 0.65) **	0.43 (0.22 to 0.84) *	0.72 (0.37 to 1.39)
Serious problems with parents			
Zero occasions	Reference	Reference	Reference
1 or more occasion	1.60 (0.94 to 2.72)	1.28 (0.89 to 1.85)	1.62 (1.11 to 2.36) *
Serious problems with friends			
Zero occasions	Reference	Reference	Reference
1 or more occasion	1.62 (1.01 to 2.61) *	1.56 (1.11 to 2.19) *	2.65 (1.86 to 3.76) **
Friends drink			
Most/all	Reference	Reference	Reference
None/a few/some	0.40 (0.20 to 0.77) **	0.53 (0.21 to 1.35)	0.49 (0.22 to 1.10)
Friends get drunk			
Most/all	Reference	Reference	Reference
None/a few/some	0.92 (0.57 to 1.50)	0.27 (0.18 to 0.43) **	0.50 (0.33 to 0.75) **
Older siblings drink			
Yes	Reference	Reference	Reference
No	0.54 (0.24 to 1.22)	0.66 (0.28 to 1.55)	0.83 (0.36 to 1.93)
Older siblings get drunk			
Yes	Reference	Reference	Reference
No	0.71 (0.37 to 1.39)	0.61 (0.33 to 1.13)	0.56 (0.31 to 1.03)

^1^ significant results * for *p* < 0.05 & ** for *p* < 0.01; † related to the Danish grading system.

## Supplementary Materials

The following are available online at http://www.mdpi.com/1660-4601/15/8/1671/s1, Table S1: Univariable analysis. Factors associated with alcohol consumption, odds ratios and 95% confidence intervals, Table S2: Multivariable gender-separated analysis. Factors associated with alcohol consumption, odds ratios and 95% confidence intervals.
