# Supplementary material for: Understanding Educational and Psychosocial Factors Associated with Alcohol Use among Adolescents in Denmark; Implications for Health Literacy Interventions
Source: Int J Environ Res Public Health. 2018 Aug 6;15(8):1671. doi: 10.3390/ijerph15081671 (PMC6121249; doi:10.3390/ijerph15081671)
Supplement: Supplementary file 1 [file ijerph-15-01671-s001.pdf]

Supplementary materials

Table S1. Univariable analysis. Factors associated with alcohol consumption, odds ratios and 95% confidence intervals.

	Drinking any alcohol during the last 30 days	Been intoxicated during the last 30 days	Problems because of own alcohol use during the last 12 months
	OR (95% CI)	OR (95% CI)	OR (95% CI)
Gender			
male	reference	reference	reference
female	.91 (.75 to 1.10)	.94 (.80 to 1.11)	.91 (.78 to 1.07)
School performance†			
6-8.9	reference	reference	reference
>9	.63 (.50 to .80)**	.74 (.60 to .92)**	.51 (.41 to .64)**
<6	.83 (.63 to 1.10)	1.17 (.93 to 1.47)	1.34 (1.07 to 1.67)*
Father's education			
Medium long or higher education	reference	reference	reference
Upper secondary education	1.03 (.77 to 1.37)	.997 (.79 to 1.26)	1.10 (.87 to 1.38)
Lower secondary education or less	1.13 (.84 to 1.50)	1.11 (.88 to 1.40)	1.37 (1.09 to 1.72)**
Mother's education			
Medium long or higher education	reference	reference	reference
Upper secondary education	1.20 (0.94 to 1.55)	.94 (.77 to 1.14)	1.04 (.85 to 1.27)
Lower secondary education or less	1.05 (0.76 to 1.44)	.90 (.69 to 1.17)	1.40 (1.09 to 1.82)*
Wealth			
About the same	reference	reference	reference
Better off	1.10 (.89 to 1.36)	1.28 (1.07 to 1.52)**	1.20 (1.01 to 1.43)*
Less well off	.87 (.63 to 1.22)	1.09 (.82 to 1.45)	1.63 (1.24 to 2.15)**
Pos. alcohol expectancies (things happen to you personally)			
Likely	reference	reference	reference
Unsure / Unlikely	.13 (.10 to .19)**	.07 (.03 to .14)**	.08 (.04 to .14)**
Neg. alcohol expectancies (things happen to you personally)			
Unsure / Unlikely	reference	reference	reference
Likely	1.92 (1.56 to 2.36)**	3.08 (2.50 to 3.80)**	3.57 (2.91 to 4.40)**
Belief risk			
Risk	reference	reference	reference
No risk or don't know	.45 (.29 to .69)**	.68 (.44 to 1.05)	.86 (.56 to 1.30)
Satisfied health			
Satisfied	reference	reference	reference
Not satisfied or Neither nor	1.03 (.78 to 1.36)	1.26 (1.01 to 1.57)*	1.60 (1.28 to 1.99)**
Satisfied themselves			
Satisfied	reference	reference	reference
Not satisfied or Neither nor	0.96 (.77 to 1.21)	1.18 (.98 to 1.42)	1.19 (.99 to 1.43)
Parents know where Saturday			
Know always / quiet often	reference	reference	reference
Know sometimes / Usually don't know	2.07 (1.22 to 3.52)**	2.89 (2.02 to 4.14)**	5.18 (3.44 to 7.80)**
Satisfied relationship mother			
Satisfied	reference	reference	reference
Not satisfied or Neither nor	1.07 (.79 to 1.47)	1.59 (1.24 to 2.03)**	2.37 (1.84 to 3.05)**
Satisfied relationship father			
Satisfied	reference	reference	reference
Not satisfied or Neither nor	1.03 (.79 to 1.34)	1.61 (1.30 to 1.99)**	2.11 (1.70 to 2.62)**
Satisfied relationship friends			
Satisfied	reference	reference	reference
Not satisfied or Neither nor	.47 (.34 to .65)**	.73 (.53 to 1.01)	0.92 (.68 to 1.24)
Serious problems with parents			
Zero occasions	reference	reference	reference
1 or more occasion	1.74 (1.37 to 2.20)**	1.97 (1.65 to 2.35)**	3.56 (2.96 to 4.27)**
Serious problems with friends			
Zero occasions	reference	reference	reference
1 or more occasion	1.66 (1.33 to 2.07)**	1.82 (1.54 to 2.15)**	3.94 (3.31 to 4.69)**

Friends drink			
Most / All	reference	reference	reference
None / A few / Some	.21 (.16 to .28)**	.25 (.17 to .35)**	.30 (.21 to .41)**
Friends get drunk			
Most / All	reference	reference	reference
None / A few / Some	.35 (.29 to .43)**	.26 (.21 to .33)**	.37 (.31 to .45)**
Older siblings drink			
Yes	reference	reference	reference
No	.34 (.24 to .48)**	.41 (.28 to .60)**	.46 (.32 to .66)**
Older siblings get drunk			
Yes	reference	reference	reference
No	.40 (.30 to .53)**	.41 (.31 to .55)**	.44 (.33 to .58)**

significant results * for p< 0.05 & ** for p < 0.01; †related to the Danish grading system

Table S2. Multivariable gender-separated analysis. Factors associated with alcohol consumption, odds ratios and 95% confidence intervals.

	**Drinking any alcohol during the last 30 days**	**Been intoxicated during the last 30 days**	**Problems because of own alcohol use during the last 12 month**
	OR (95% CI)	OR (95% CI)	OR (95% CI)
School performance†			
6-8.9 (average)	reference	reference	reference
>9 (above average) – girls	.41 (.23 to .74)**	.71 (.43 to 1.18)	.50 (.29 to .84)*
<6 (below average) – girls	.75 (.27 to 2.05)	1.61 (.76 to 3.40)	.95 (.43 to 2.11)
>9 (above average) – boys	1.06 (.50 to 2.26)	1.22 (.67 to 2.23)	.68 (.37 to 1.27)
<6 (below average) – boys	2.81 (.94 to 8.34)	1.41 (.75 to 2.63)	1.45 (.76 to 2.76)
Father's education			
Medium long or higher education	reference	reference	reference
Upper secondary education – girls	.69 (.34 to 1.40)	1.63 (.94 to 2.85)	1.42 (.80 to 2.53)
Lower secondary education or less – girls	.84 (.40 to 1.73)	1.26 (.70 to 2.27)	2.01 (1.10 to 3.66)*
Upper secondary education – boys	.70 (.32 to 1.54)	.73 (.40 to 1.33)	.92 (.51 to 1.69)
Lower secondary education or less – boys	.97 (.42 to 2.24)	.73 (.39 to 1.35)	.88 (.47 to 1.65)
Mother's education			
Medium long or higher education	reference	reference	reference
Upper secondary education – girls	1.34 (.69 to 2.62)	.53 (.32 to .90)*	.82 (.48 to 1.40)
Lower secondary education or less – girls	.63 (.27 to 1.43)	.46 (.23 to .90)*	.76 (.38 to 1.52)
Upper secondary education – boys	1.38 (.68 to 2.79)	1.47 (.86 to 2.50)	2.02 (1.18 to 3.45)*
Lower secondary education or less – boys	1.44 (.53 to 3.90)	1.47 (.73 to 2.96)	2.86 (1.39 to 5.88)**
Wealth			
About the same	reference	reference	reference
Better off – girls	1.29 (.73 to 2.26)	1.33 (.85 to 2.08)	1.29 (.81 to 2.04)
Less well off – girls	.38 (.14 to 1.04)	.83 (.33 to 2.11)	1.64 (.64 to 4.23)
Better off – boys	1.06 (.56 to 2.01)	1.41 (.86 to 2.31)	1.13 (.69 to 1.86)
Less well off – boys	1.45 (.40 to 5.26)	1.00 (.39 to 2.58)	.64 (.25 to 1.66)
Pos. alcohol expectancies (things happen to you personally)			
Likely	reference	reference	reference
Unsure / Unlikely – girls	.12 (.35 to .40)**	.18 (.02 to 1.47)	.12 (.01 to 1.12)
Unsure / Unlikely – boys	.44 (.17 to 1.12)	.27 (.06 to 1.25)	.17 (.37 to .80)*
Neg. alcohol expectancies (things happen to you personally)			
Unsure / Unlikely	reference	reference	reference
Likely – girls	.72 (.38 to 1.40)	1.95 (1.11 to 3.43)*	2.10 (1.18 to 3.75)*
Likely – boys	1.92 (1.03 to 3.57)*	2.65 (1.54 to 4.56)**	3.93 (2.28 to 6.80)**
Belief risk			
Risk	reference	reference	reference
No risk or don't know – girls	.98 (.04 to 23.46)	.38 (.03 to 4.65)	1 (omitted, collinearity)
No risk or don't know – boys	.12 (.04 to .25)**	.54 (.18 to 1.60)	.57 (.19 to 1.70)
Satisfied health			
Satisfied	reference	reference	reference
Not satisfied or Neither nor – girls	.63 (.30 to 1.31)	.70 (.39 to 1.25)	.93 (.50 to 1.72)
Not satisfied or Neither nor – boys	.77 (.29 to 2.01)	1.28 (.59 to 2.77)	.98 (.44 to 2.19)
Satisfied themselves			
Satisfied	reference	reference	reference

Not satisfied or Neither nor – girls	1.52 (.82 to 2.83)	1.44 (.91 to 2.28)	.98 (.60 to 1.59)
Not satisfied or Neither nor – boys	.57 (.24 to 1.38)	1.26 (.61 to 2.61)	1.32 (.63 to 2.76)
Satisfied mother			
Satisfied	reference	reference	reference
Not satisfied or Neither nor – girls	1.13 (.38 to 3.35)	1.25 (.60 to 2.63)	1.85 (.83 to 4.14)
Not satisfied or Neither nor – boys	1.26 (.33 to 4.81)	.82 (.35 to 1.96)	1.74 (.69 to 4.41)
Satisfied father			
Satisfied	reference	reference	reference
Not satisfied or Neither nor – girls	1.29 (.56 to 2.95)	1.58 (.87 to 2.89)	1.13 (.60 to 2.13)
Not satisfied or Neither nor – boys	.46 (.18 to 1.19)	.79 (.36 to 1.73)	.92 (.42 to 2.03)
Satisfied friends			
Satisfied	reference	reference	reference
Not satisfied or Neither nor – girls	.39 (.16 to .95)*	.50 (.22 to 1.13)	.70 (.30 to 1.62)
Not satisfied or Neither nor – boys	.26 (.08 to .85)*	.34 (.10 to 1.17)	.76 (.24 to 2.39)
Parents know where Saturday			
Know always / quiet often	reference	reference	reference
Know sometimes / Usually don't know – girls	.99 (.11 to 8.89)	.81 (.23 to 2.89)	.80 (.18 to 3.60)
Know sometimes / Usually don't know – boys	.75 (.17 to 3.25)	.78 (.28 to 2.21)	1.18 (.36 to 3.86)
Serious problems with parents			
Zero occasions	reference	reference	reference
1 or more occasion – girls	1.83 (.87 to 3.82)	.94 (.55 to 1.59)	1.53 (.90 to 2.62)
1 or more occasion – boys	1.73 (.72 to 4.13)	2.09 (1.19 to 3.67)*	1.94 (1.09 to 3.47)*
Serious problems with friends			
Zero occasions	reference	reference	reference
1 or more occasion – girls	1.78 (.96 to 3.30)	2.19 (1.37 to 3.51)**	3.36 (2.09 to 5.40)**
1 or more occasion – boys	1.37 (.60 to 3.12)	1.02 (.60 to 1.73)	2.05 (1.18 to 3.57)*
Friends drink			
Most / All		reference	reference
None A few / Some – girls	.30 (.11 to .83)*	.66 (.17 to 2.52)	.51 (.15 to 1.80)
None / A few / Some – boys	.51 (.19 to 1.41)	.47 (.12 to 1.83)	.43 (.15 to 1.27)
Friends get drunk			
Most / All	reference	reference	reference
None / A few / Some – girls	.70 (.36 to 1.36)	.27 (.15 to .51)**	.40 (.22 to .72)**
None A few / Some – boys	1.05 (.48 to 2.27)	.26 (.13 to .53)**	.58 (.31 to 1.09)
Older siblings drink			
Yes	reference	reference	reference
No – girls	.23 (.05 to .95)*	.90 (.22 to 3.77)	1.21 (.27 to 5.42)
No – boys	.83 (.28 to 2.44)	.48 (.16 to 1.44)	.63 (.22 to 1.81)
Older siblings get drunk			
Yes	reference	reference	reference
No – girls	1.20 (.36 to 3.93)	.53 (.19 to 1.54)	.29 (.10 to .89)*
No – boys	.52 (.22 to 1.24)	.69 (.31 to 1.50)	.81 (.38 to 1.74)

[1] significant results * for p< 0.05 & ** for p < 0.01; †related to the Danish grading system
